# A prediction model based on DNA methylation biomarkers and radiological characteristics for identifying malignant from benign pulmonary nodules

**DOI:** 10.1186/s12885-021-08002-4

**Published:** 2021-03-10

**Authors:** Wenqun Xing, Haibo Sun, Chi Yan, Chengzhi Zhao, Dongqing Wang, Mingming Li, Jie Ma

**Affiliations:** 1 Department of Thoracic Surgery, Affiliated Cancer Hospital of Zhengzhou University, Henan Cancer Hospital, Zhengzhou, Henan China; 2 Department of Molecular Pathology, Affiliated Cancer Hospital of Zhengzhou University, Henan Cancer Hospital, No 127, Dongming Road, Zhengzhou, 450008 Henan China; 3 Henan Key Laboratory of Molecular Pathology, Zhengzhou, Henan China; 4 Excellen Medical Technology Co., Ltd., Beijing, China

**Keywords:** CT, DNA methylation, Biomarkers, Lung cancer, Pulmonary nodules

## Abstract

**Background:**

Lung cancer remains the leading cause of cancer deaths across the world. Early detection of lung cancer by low-dose computed tomography (LDCT) can reduce the mortality rate. However, making a definitive preoperative diagnosis of malignant pulmonary nodules (PNs) found by LDCT is a clinical challenge. This study aimed to develop a prediction model based on DNA methylation biomarkers and radiological characteristics for identifying malignant pulmonary nodules from benign PNs.

**Methods:**

We assessed three DNA methylation biomarkers (*PTGER4*, *RASSF1A,* and *SHOX2*) and clinically-relevant variables in a training cohort of 110 individuals with PNs. Four machine-learning-based prediction models were established and compared, including the K-nearest neighbors (KNN), random forest (RF), support vector machine (SVM), and logistic regression (LR) algorithms. Variables of the best-performing algorithm (LR) were selected through stepwise use of Akaike’s information criterion (AIC). The constructed prediction model was compared with the methylation biomarkers and the Mayo Clinic model using the non-parametric approach of DeLong et al. with the area under a receiver operator characteristic curve (AUC) analysis.

**Results:**

A prediction model was finally constructed based on three DNA methylation biomarkers and one radiological characteristic for identifying malignant from benign PNs. The developed prediction model achieved an AUC value of 0.951 in malignant PNs diagnosis, significantly higher than the three DNA methylation biomarkers (0.912, 95% CI:0.843–0.958, *p* = 0.013) or Mayo Clinic model (0.823, 95% CI:0.739–0.890, *p* = 0.001). Validation of the prediction model in the testing cohort of 100 subjects with PNs confirmed the diagnostic value.

**Conclusion:**

We have shown that integrating DNA methylation biomarkers and radiological characteristics could more accurately identify lung cancer in subjects with CT-found PNs. The prediction model developed in our study may provide clinical utility in combination with LDCT to improve the over-all diagnosis of lung cancer.

**Supplementary Information:**

The online version contains supplementary material available at 10.1186/s12885-021-08002-4.

## Background

Lung cancer is the second most common cancer globally and the leading cause of cancer mortality worldwide [1]. In 1987, it surpassed breast cancer as the leading cause of cancer-related deaths of women. By2020, Lung cancer is expected to account for 22% of all female cancer deaths and 23% of all male cancer deaths [1].

The exceptional high mortality of lung cancer can be attributed to a high degree by late diagnosis. The 5-year survival rate of lung cancer is only 15–19% at all stages. Outcomes can be significantly better at an early-stage diagnosis, especially for stage I, the 5-year survival rate can increase up to 81–85% [2, 3]. Thus, it seems reasonable to improve lung cancer screening at earlier stages. Low-dose computed tomography (LDCT) is widely accepted as a reliable screening tool for lung cancer early detection. The National Lung Screening Trial (NLST) reported that LDCT decreases the mortality rate by 20% in high-risk people [4, 5]. However, Pulmonary nodules (PNs) are encountered with increased frequency in asymptomatic individuals due to the widespread use of LDCT. High false-positive rates and overdiagnosis limited the diagnostic accuracy of LDCT screening. The National Lung Screening Trial showed that in heavy smokers, the positive rate of indeterminate PNs detected by LDCT was 24.2%; however, 96.4% of these PNs were ultimately confirmed to be false positives over the three rounds of screening [5].

Currently, to predict the malignancy probability of PNs found by LDCT, a series of examination techniques have been proposed, including non-invasive and invasive approaches [6]. Each approach has advantages and disadvantages. Noninvasive approaches include follow-up with positron emission tomography, LDCT, or magnetic resonance imaging for up to 2 years to determine whether it is a benign lesion. These non-invasive approaches often result in unnecessary radiation exposure, anxiety, procedures, and additional cost for subjects with benign lesions. A CT-guided transthoracic needle biopsy can establish a specific benign or malignant diagnosis but is invasive, potentially risky, and sometimes non-diagnostic [7]. Thus, it is clinically significant to develop new approaches to accurately identify patients with malignant from benign PNs safely and cost-effectively.

Analysis of lung tumor-associated molecular changes in body fluids may provide a safe and cost-effective approach for detecting lung cancer. DNA methylation is a relatively stable biochemical modification; it can be detected not only from tissue but also in serum and plasma [8]. Assessment of DNA methylation in plasma offers a potentially cost-effective method in discriminating malignant from benign PNs. Prostaglandin E receptor 4 gene (*PTGER4*), ras association domain family 1A (*RASSF1A*), and short stature homeobox gene two (*SHOX2*) methylation have been separately identified as valuable biomarkers for lung cancer diagnosis in several research studies [9–12]. However, investigating whether the three methylation biomarkers are useful in distinguishing lung cancer among individuals with LDCT-detected PNs has hardly been reported.

Previous studies also showed that, based on subjects’ demographic characteristics and radiological features of PNs on CT images, the constructed predictive models could identify malignant from benign PNs [13–16]. For example, Swensen et al. developed a Mayo Clinic model based on six independent predictors (patients’ age, smoking history, cancer history, nodule diameter, upper lobe position, and spiculation), which had an AUC of 0.83 for the diagnosis of malignant PNs [13]. Gould et al. established another prediction model, which yielded 0.78 AUC based on age, smoking history, nodule diameter, and smoking cessation [14, 15]. Recently, McWilliams et al. also developed two similar prediction models, with AUCs of 0.89–0.91 [16]. Although these clinical/radiological characteristics-based models are promising in identifying malignant PNs, the diagnostic accuracy still need improvement.

Considering the complex tumor microenvironment and clonal selection in lung cancer development, using circulating biomarkers alone or clinical/radiological factors alone might not have sufficient diagnostic accuracy for lung cancer. We aimed to investigate if combining DNA methylation biomarkers with clinical/radiological characteristics could more efficiently distinguish malignant from benign lung nodules detected by LDCT.

## Materials and methods

### Ethics

We enrolled participants in Henan Cancer Hospital, the Affiliated Cancer Hospital of Zhengzhou University. All participants signed the informed consent before blood collection, and they were informed of the usage of plasma and the test results. The current study has been received approval from the Medical Ethics Committee of Henan Cancer Hospital (2018157).

### Patient cohorts and study design

The participants were enrolled with lung nodules newly detected in Henan Cancer Hospital from January 2019 to December 2019. We obtained blood samples from all subjects who met the selection criteria. The inclusion criteria were: (I) subjects detected pulmonary nodules on CT scans. (II) LDCT-derived nodule diameter between 4 and 35 mm; (III) the participants’ clinical information should be complete. The exclusion criteria were: (I) pregnancy or lactation; (II) current pulmonary infection; (III) surgery within 6 months; (IV) radiotherapy within 1 year; and (V) life expectancy of < 1 year.

For the selected patients, CT examinations were performed at our institution with the Revolution CT (General Electric Medical Systems, Milwaukee, Wisconsin, USA) or the Brilliance iCT (Philips Healthcare, Best, The Netherlands) using a tube voltage of 120 kV and a current of 200 mA. The target lesion was reconstructed with the following standard reconstruction parameters: slice thickness, 1.0 mm; increment, 1 mm; pitch, 1.078; a field of view, 15 cm; and a matrix of 512 × 512. We collected the general characteristics and nodule radiographic characteristics of participants from the hospital information system. General characteristics included age, gender, smoking behavior (smoking status, pack-years, and the number of years since quitting), and cancer history. Nodule radiographic characteristics comprised the maximum transverse size; location; and nodule type (nonsolid or ground-glass opacity, perifissural, part-solid, solid, and spiculation). The radiographic characteristics of PNs were obtained from the radiology report, documentation provided by an attending pulmonologist or thoracic surgeon, and by review of imaging by the research team. In the event of disagreement, the interpretation of the research team was used. Malignant or benign diagnosis of PNs was verified based on the pathologic examination of tissues obtained via surgery or biopsy. The surgical pathologic staging was determined based on the TNM guidelines classification criteria [17]. According to the World Health Organization classification to determine the histopathologic classification [18].

### Sample collection and storage

Plasma samples were collected from outpatients and inpatients of Henan Cancer Hospital, and the sample information was recorded in sample collection forms. Five millilitre of peripheral blood from the subject was drawn in a 5-ml K_2_EDTA anticoagulant tube (BD biosciences, Franklin Lakes, NJ, USA). The plasma sample’s storage and transportation followed the instructions of the Nucleic Acid Extraction Reagent (Excellen Medical Technology Co., Ltd.).

### DNA isolation and bisulfite conversion

Blood samples were collected before surgery, anesthesia, and adjuvant therapy. The collected specimens were processed within 4 h by centrifuging at 3000 g for 10 min at 4 °C. Then, we transferred the collected plasma to a new tube and stored at − 80 °C until use. DNA was extracted from plasma using the Nucleic Acid Extraction Reagent (Excellen Medical Technology Co., Ltd.) according to the instructions. In Brief, circulating DNA was extracted from 2 mL of plasma utilizing magnetic beads, then converted the unmethylated cytosine residue to uracil residue in DNA by a bisulfite reaction. After further purification, bisulfite-converted DNA (bisDNA) was eluted in 35 μL and ready for real-time PCR use.

### DNA methylation analysis

DNA methylation analysis was performed according to the diagnostic kit’s instructions (Excellen Medical Technology Co., Ltd.). The eluted DNA was used as a template for fluorescent real-time PCR. Each PCR reaction mixture has a total reaction volume of 25 μL, including 12.5 μL reaction buffer, 2.5 μL primer mix, and 10 μL eluted DNA. Fluorescence PCR amplifications were performed on 96-well plates of Applied Biosystems 7500 Fast Real-Time PCR Systems. Each sample was carried out in triplicate. In addition to subject DNA samples, each plate also included positive controls (in vitro methylated leukocyte DNA), negative controls (normal leukocyte DNA or DNA from a known unmethylated cell line), and water blanks. The thermal profile for amplification reactions was 98 °C for 5 min, followed by 45 cycles at 95 °C for 10 s and 63 °C for 5 s to 58 °C for 30 s. In the PCR reaction, the primers and probes were designed to amplify the methylated sequences preferentially. During the PCR process, the methylated target sequence can be exclusively identified from unmethylated DNA. Increased inflorescent emission of the reporter dye can be detected on fluorescence channels of FAM, HEX, Texas Red, and CY5. The resulting data were analyzed by Applied Biosystems 7500 Fast Real-Time PCR System Sequence Detection Software v1.4.1.

All samples were within the range of the assay of sensitivity and reproducibility based on the amplification of internal reference standard [threshold cycle (Ct) value for β-Actin (ACTB)]. We calculated the 2^-ΔCT^ for each methylation detection replicate comparing it to the mean Ct for ACTB, the average value of triplicates of selected gene divided by the average value of triplicates of ACTB. For some samples replicates with the extremely low levels of DNA methylation in plasma, a Ct of 45 was used, creating a near-zero value for 2^-ΔCT^. Lung Cancer Analysis software v2.2 (Excellen Medical Technology Co., Ltd.) analyzes the PCR output, calculating a composite score, reflecting the overall methylation level in the assay’s markers.

### Statistical analysis

Adapted from Han et al. [19], we applied four well-established machine-learning algorithms to predict a malignant or benign nodule (as a binary variable). The K-nearest neighbors (KNN), random forest (RF), support vector machine (SVM), and logistic regression (LR) algorithms used the DNA Methylation and clinically-relevant variables as candidate features. We evaluated the performance of classifiers through fourfold cross-validation within the training set. In detail, we randomly divided the training set into four equal portions; then, during each of the four iterations, we first applied the 3/4 of the training data trained the classifiers (500 trees for RF, the radial kernel for SVM, other parameters set by default). Next, we applied the trained classifiers to the remaining 1/4 of the training data for prediction. The predictions from all four iterations were combined and compared with the truth, then a receiver operator characteristic curve (ROC) was created and the area under the curve (AUC) was computed to evaluate the prediction capability for each model separately. Finally, we applied the classifier trained from the whole training set to an independent sample to independently validate the predictive power.

The variables for the final model of binomial logistic regression were selected through stepwise use of Akaike’s information criterion (AIC). Then the selected variables were used to fit an ordinary logistic regression model and estimate the regression coefficients. The final constructed prediction model was validated in an independent sample for identifying malignant PNs.

The primary endpoint was the diagnostic accuracy for malignant PNs. We assessed each model’s diagnostic accuracy by calculating the area under the ROC curve (AUC) and 95% confidence intervals (CI). The non-parametric approach of DeLong et al. was used to compare the performance of the prediction model with that of the plasma biomarkers and the Mayo Clinic model [20]. The prediction model was developed in a cohort’s training set and blindly validated in an additional set of subjects by comparing the calculated results with the final clinical diagnosis and the AUCs. We conducted a power analysis for the comparison between performance in the Mayo model versus our constructed prediction model with power (1 – β) set at 0.8 and α = 0.05. Based on published data [13], the expected AUC value of the Mayo model for identifying PNs was defined as 0.85. The analysis yielded a required sample size of 91 participants for detection 10% difference, estimated by the formula published previously [21]. R version 3.3.2 (The R Foundation for Statistical Computing) and MedCale Statistics were used for all analyses. *P* values < 0.05 were considered to indicate statistical significance.

## Results

### Clinical characteristics of subjects

Altogether, we recruited 210 subjects, of which 120 were diagnosed with malignant PNs, 90 nodules were diagnosed with benign. The subjects were divided into a training cohort and a validation cohort by enrollment time. The initial series of 110 cases and controls were used for training and the subsequent series of 100 was used for validation. For each patient, only the largest nodule confirmed by histopathology was chosen for analysis. In the training cohort, 110 nodules, of which 63 were malignant, and in the validated cohort, 100 nodules, of which 57 were malignant (Table 1). Among persons with nodules, the rates of cancer in the two data sets were 57.3 and 57.0%, respectively. Subjects with lung cancer were generally older than subjects with benign nodules (58 vs 55 years). Of the subjects, 63 (57.3%) were male, and 69 (62.7%) were non-smokers. The 63 subjects with malignant PNs were diagnosed with adenocarcinomas (*n* = 37), squamous cell carcinomas (*n* = 14), small cell lung cancer (*n* = 8) and, unclassified lung cancer patients (*n* = 4). The LC patients consisted of 17 stage I, 21 stage II, and 25 stage III to IV cases. One hundred subjects with PNs were used as a validated cohort to confirm the prediction model for the differentiation of malignant from benign PNs. The cohort consisted of 57 subjects with malignant PNs (LC) and 43 subjects with benign PNs (Table 2). Of the patients with malignant PNs, 32 were diagnosed with adenocarcinomas, 14 were diagnosed with squamous cell carcinomas, 2 were diagnosed with small cell lung cancer, and 9 were unclassified lung cancer patients. The demographic and clinical parameters, including detailed information about the two cohorts’ nodule characteristics, are shown in Tables 1 and 2, respectively.

**Table 1:** Subjects’ Characteristics of Training Study

Characteristics	Subjects with Malignant PNs (***n*** = 63)	Subjects with Benign PNs (***n*** = 47)
**Clinical**		
**Age (Years)**		
Median Age	58	55
Age Range	36–77	26–70
**Sex**		
Male	36	27
Female	27	20
**Smoking history**		
Non-smoker	30	39
Ex-smoker	12	3
Current smoker	21	5
**Smoking pack-years**		
Mean Pack-years (Smokers only)	36.52	20
Years quit (Smoker sonly)	8.9	3
**Histology subtype**		
Adenocarcinoma	37	
Squamous cell carcinoma	14	
Small cell lung cancer	8	
Other	4	
**Stage**		
I	17	
II	21	
III-IV	25	
**Radiological**		
**Nodule size (mm)**	21.46 (SD 10.52)	11.89 (SD 6.81)
**Nodule location**		
Left lower lobe	9	6
Left upper lobe	17	12
Right lower lobe	15	15
Right middle lobe	3	4
Right upper lobe	19	10
**Nodule type (number)**		
Nonsolid or ground-glass opacity	17	13
Perifissural	6	5
Part-solid	9	8
Solid	13	11
Spiculation	18	10

*PN* pulmonary nodule, *SD* standard deviation

**Table 2:** Subjects’ Characteristics of Validation Study

Characteristics	Subjects with Malignant PNs (***n*** = 57)	Subjects with Benign PNs (***n*** = 43)
**Clinical**		
**Age (Years)**		
Median Age	62	54
Age Range	38–78	27–72
**Sex**		
Male	39	29
Female	18	14
**Smoking history**		
Non-smoker	25	30
Ex-smoker	12	4
Current smoker	20	9
**Smoking pack years**		
Mean Pack-years (Smokers only)	40.77	21.64
Years quit (Smoker sonly)	7.35	3.75
**Histology subtype**		
Adenocarcinoma	32	
Squamous cell carcinoma	14	
Small cell lung cancer	2	
Other	9	
**Stage**		
I	18	
II	20	
III-IV	19	
**Radiological**		
**Nodule size (mm)**	21.83 (SD 10.88)	11.22 (SD 7.56)
**Nodule location**		
Left lower lobe	12	10
Left upper lobe	12	10
Right lower lobe	11	11
Right middle lobe	5	4
Right upper lobe	17	8
**Nodule type (number)**		
Nonsolid or ground-glass opacity	17	13
Perifissural	8	6
Part-solid	9	7
Solid	13	10
Spiculation	10	7

*PN* pulmonary nodule, *SD* standard deviation

### Diagnostic accuracy of the three methylation biomarkers for identifying malignant PNs

**Fig. 1:**
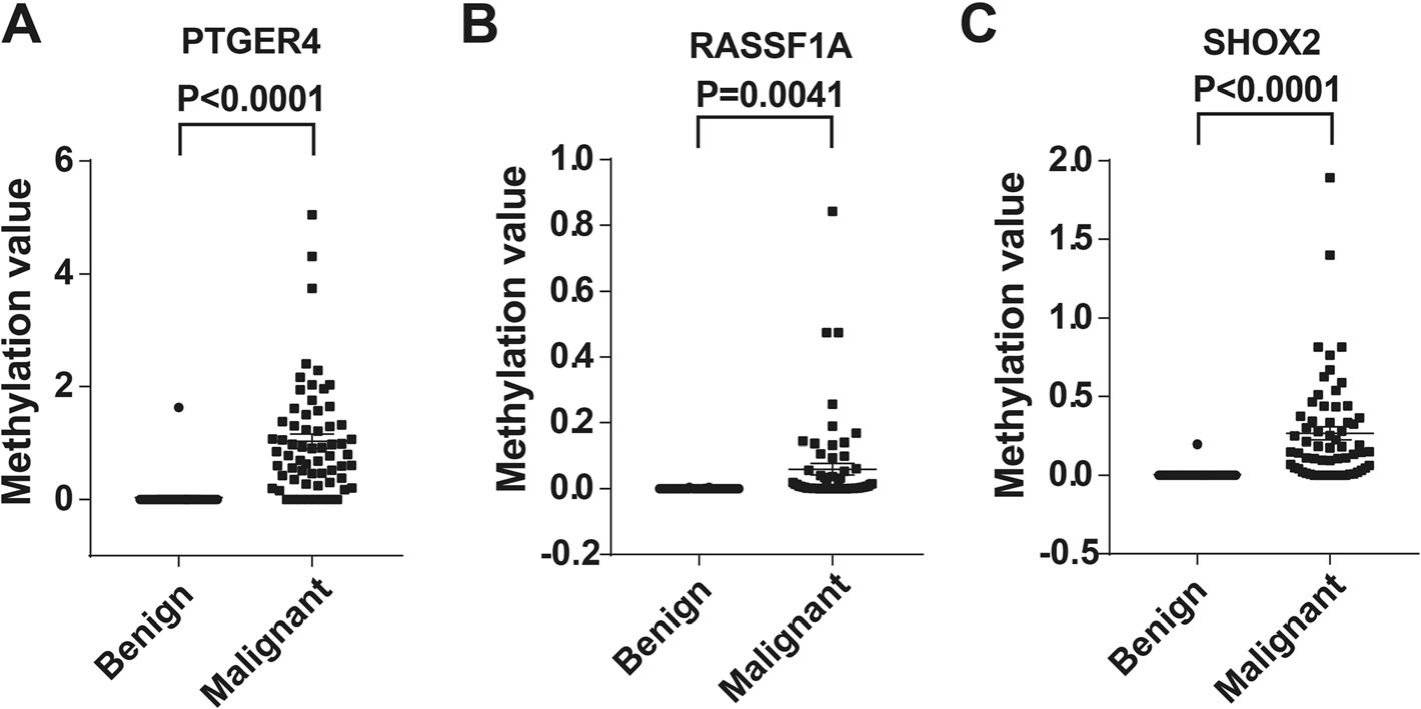
Comparison of the studied DNA methylation expressions in patients with benign PNs, and patients with malignant PNs in a training cohort. Scatter plots show the distribution of relative normalized methylation values for each of the 3 genes determined by q-PCR. The paired t-test was performed

To determine the diagnostic values of the three methylation biomarkers, we quantitative analysis of promoter methylation in the plasma DNA samples from the training cohort of 110 subjects using the diagnostic kit for the methylated gene of lung cancer (Excellen Medical Technology Co., Ltd.). Plasma expression level for each methylation biomarker was compared between two groups of subjects in the training set. As shown in Fig. 1, three methylation biomarkers displayed higher plasma expression levels in patients with malignant PNs compared to individuals with benign PNs (All *P* < 0.01). The three methylation biomarkers present potential plasma biomarkers for identifying malignant PNs.

**Fig. 2:**
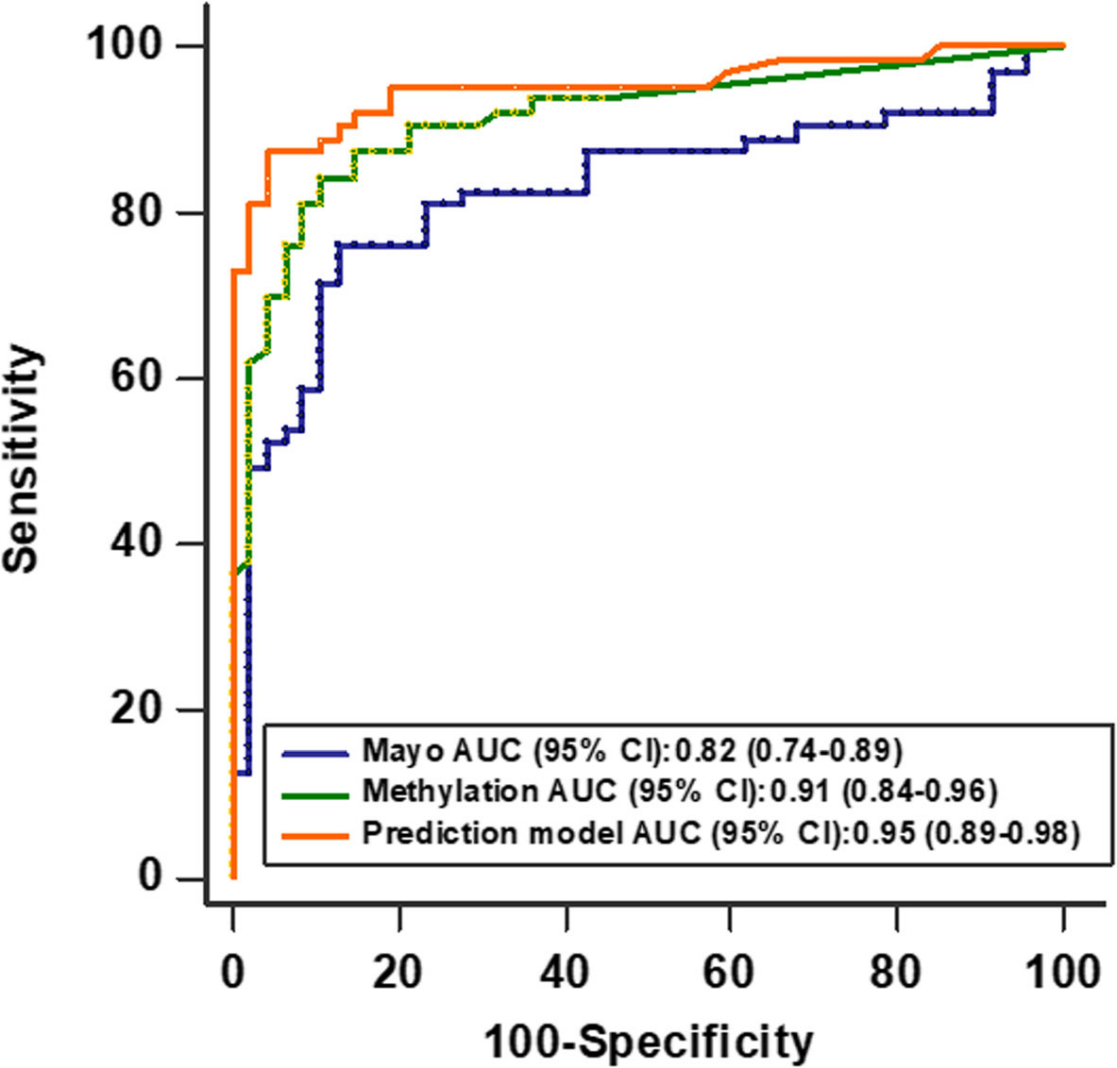
Receiver-operator characteristic (ROC) curve analysis of the three models in a training cohort. The area under the ROC curve (AUC) for each model conveys its accuracy for diagnosing malignant PNs. The prediction model produced a higher AUC value for identifying malignant PNs comparing with the panel of the three DNA methylation biomarkers and the Mayo Clinic model

We further performed receiver operating characteristic (ROC) curve analysis to evaluate the capability of using the three methylation biomarkers to discriminate patients with malignant PNs from patients having benign PNs. As shown in Fig. 2, the three DNA methylation used in combination yielded 0.912 AUC in identifying malignant from benign PNs. No statistically significant association was observed between the logistic model with subjects’ age, gender, and smoking history (all *p* > 0.05).

### Developing a prediction model based on the methylation biomarkers and radiographic features of PNs for distinguishing malignant from benign PNs

Although use of the three DNA methylation showing promise with an AUC value of 0.912, it is not sufficient for identifying malignant PNs in the clinic. To improve the diagnostic accuracy for malignant PNs, we applied a rigorous machine-learning approach to assess the combined use of methylation biomarkers and all clinically-relevant variables in classifying PNs. First, within the training set, we applied four well-established machine-learning algorithms K-nearest neighbors (KNN), random forest (RF), support vector machine (SVM), and logistic regression (LR) and evaluated their performance based on the area under the receiver operating characteristics curve (AUC) through fourfold cross-validation. We found that the models of SVM and LR can accurately classify malignant from benign PNs with AUC of 0.92 and 0.93. Moreover, the best-performing algorithm, LR, achieved a high AUC of 0.96 on the independent test set (Table 3). These results indicated that the combined use of methylation biomarkers and clinically-relevant variables can effectively provide an independent approach to validate the classification of tumor subtypes.

**Table 3:** Accuracy and predictive value between four models

Cross Validation	Model	Sensitivity	Specificity	PPV	NPV	Accuracy	AUC
**4-fold on training cohort**	KNN	0.83	0.86	0.90	0.80	0.83	0.84
	SVM	0.89	0.85	0.89	0.86	0.87	0.92
	RF	0.88	0.85	0.89	0.85	0.87	0.91
	**RL**	**0.91**	**0.83**	**0.88**	**0.89**	**0.87**	**0.93**
**Validated in an independent cohort**	KNN	0.93	0.84	0.9	0.9	0.89	0.88
	SVM	0.93	0.93	0.91	0.91	0.93	0.96
	RF	0.91	0.93	0.89	0.89	0.92	0.95
	**RL**	**0.91**	**0.88**	**0.88**	**0.91**	**0.9**	**0.96**

*KNN* K-nearest neighbors, *SVM* support vector machine, *RF* random forest, *RL* logistic regression, *AUC* area under the curve, *PPV* positive predictive value, *NPV* negative predictive value

We next used the logistic regression model through stepwise use of Akaike’s information criterion (AIC) to select the variables for the final models. Once the AIC value no longer decreases, the stepwise regression analysis terminates and the optimal regression equation is output. Finally, the logistic regression model selected the methylation biomarkers (*p* < 0.001) and diameter of PNs (*p* < 0.001) as significant predictors for malignant PNs (See Additional file 1). Variables were presented in the prediction model by using the following formula: the probability of malignant PNs = e^x^/(1 + e^x^), where e is the base of the natural logarithm and x = − 4.433 + 1.066 × Composite score + 0.151 × Diameter of PNs. Then, we evaluated the performance of this prediction model in the training set, which produced 0.951 AUC in identifying malignant from benign PNs (Fig. 2). It has been reported that several prediction models based on PNs parameters on CT images and clinical characteristics of subjects developing to predict the probability of malignant PNs [13–16], of which the Mayo Clinic model is a commonly used one. We also applied the equation of the Mayo Clinic model: Probability of Malignancy = e^x^/(1 + e^x^), x = − 6.8272 + (0.0391 × Age) + (0.7917 × Smoking history) + (1.3388 × Cancer) + (0.1274 × Diameter) + (1.0407 × Spiculation) + (0.7838 × Upper) [13] to predict malignant PNs in the training cohort of 110 subjects, as shown in Fig. 2. The AUC value obtained by the Mayo Clinic model was 0.823, and the value was similar to the previous reports [13–15]. The AUC value of the prediction model (0.951, 95% CI:0.892–0.983) was significantly higher than the panel of the three methylation biomarkers (0.912, 95% CI: 0.843–0.958, *p* = 0.013) used alone and the Mayo Clinic model (0.823, 95% CI:0.739–0.890, *p* = 0.001).

### Validating the prediction model for identifying malignant PNs in an independent cohort

**Fig. 3:**
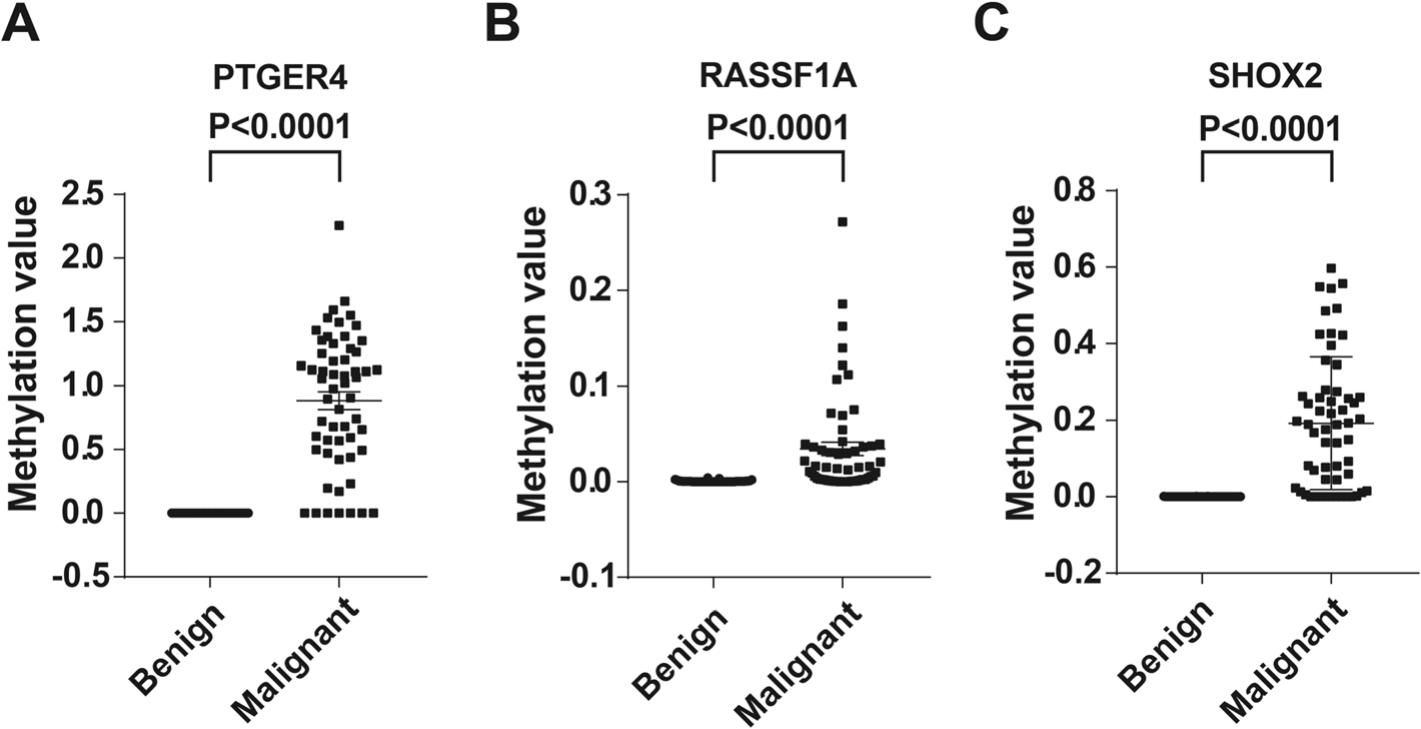
Comparison of the studied DNA methylation expressions in patients with benign PNs, and patients with malignant PNs in an independent cohort. Scatter plots show the distribution of relative normalized methylation values for each of the 3 genes determined by q-PCR. The paired t-test was performed

**Fig. 4:**
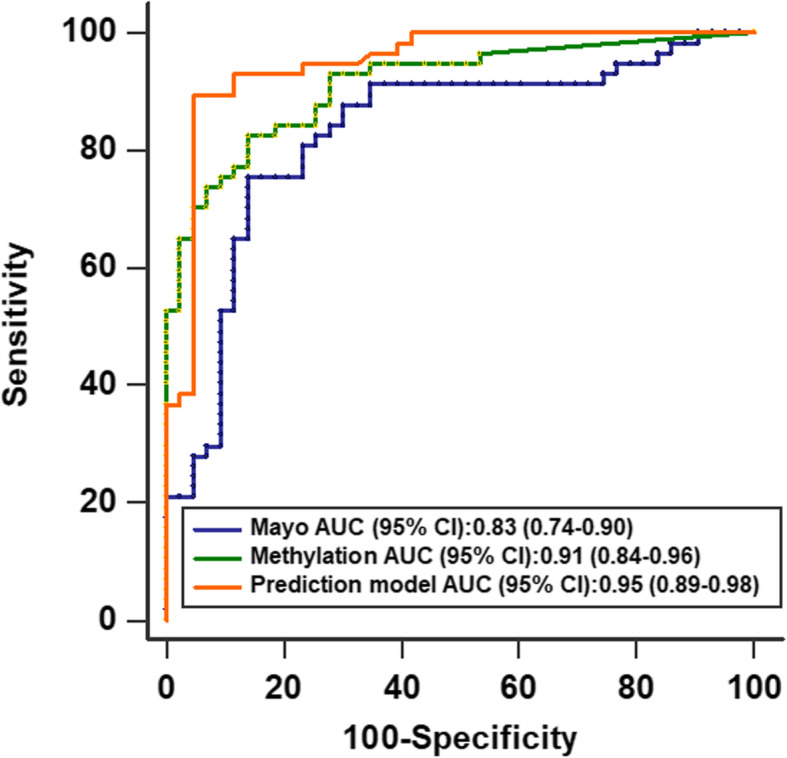
Comparison of ROC curves generated using the prediction model, panel of the three DNA methylation biomarkers, and Mayo Clinic model in an independent cohort. The prediction model produced the highest AUC value of the three models

Firstly, we confirmed the expression of the DNA methylation panel in an independent cohort. The three-gene methylation biomarkers displayed higher plasma expression levels in patients with malignant PNs compared to individuals having benign PNs (All *p* < 0.0001) (Fig. 3). The observations were in agreement with the findings observed in the above training test, which indicated that the gene methylation could be reproducibly measured. Then, we evaluated the diagnostic performance of the three models. The AUC value of the prediction model in the validated cohort (0.948) was similar to in the training cohort (0.951). As shown in Fig. 4, the AUC value of the prediction model was significantly higher than the panel of the biomarkers (0.912, 95% CI: 0.84–0.96) and the Mayo Clinic model (0.829, 95% CI: 0.94–0.90). We used the optimal cut-offs obtained in the training set to determine the prediction model’s diagnostic performance in the validated cohort. The prediction model produced a sensitivity of 89.5% and a specificity of 95.4%. Taken together, these results confirmed that the prediction model had the potential for estimating malignant PNs among individuals with CT-detected PNs.

## Discussion

Low-dose spiral computed tomography (LDCT), a reliable screening tool for early detection of lung cancer, was severely limited by its low specificity [4, 5]. LDCT dramatically increases the number of indeterminate pulmonary nodules (PNs), whereas most PNs are ultimately false positives [22]. It is clinically significant to develop new methods that can precisely identify malignant from benign PNs safely and cost-effectively.

Some clinical/radiological characteristics-based models have shown the potential to identify malignant PNs [13–15]. The finding from our present study confirmed the previous observations. However, the moderate sensitivity and specificity of these models limit the application in clinical. DNA methylation plays a vital role in tumorigenesis at an early stage [23–25]. That makes DNA methylation alterations among the most promising candidates in biomarker research. To improve the diagnostic accuracy of lung cancer, various DNA methylation biomarkers have been explored. Among them, *PTGER4, RASSF1A, and SHOX2* methylation biomarkers showed high potential in the diagnosis and prognosis of lung cancer. Kneip C et al. performed DNA methylation analysis of the *SHOX2* gene in blood, the result showed a sensitivity of 60% and specificity of 90% in the diagnosis of lung cancer [11]. Hu et al. reported that promoter hypermethylation of *RASSF1A* occurs frequently in lung cancer and is frequently found in small cell lung cancer [12]. Besides, Weiss G et al. validated that *SHOX2/PTGER4* DNA methylation marker panel could discriminate between patients with malignant and nonmalignant lung disease with an AUC value of 0.88 [9]. Inspired by these studies, we combined detection of *PTGER4*, *RASSF1A*, and *SHOX2* methylation biomarkers for estimating malignant from benign PNs in a training cohort. The three methylation biomarkers used in combination produced an AUC value of 0.912. Despite showing promise, the diagnostic accuracy also needed to be further improved. We developed a novel lung nodule risk prediction model by integrating the three DNA methylation biomarkers with one radiological variable of PNs to estimate the probability of malignancy in PNs. The prediction model has a higher AUC value than the Mayo Clinic model or the panel of biomarkers used alone. Furthermore, in an independent cohort, the prediction model’s performance validated, further confirming the tremendous potential for detecting malignant PNs. Our current findings suggested that the prediction model with three DNA methylation biomarkers and the diameter of PNs may potentially guide the management of CT screening results.

Based on the Food and Drug Administration criteria, a disease with a 5% prevalence, the screening test should have a sensitivity exceeding 95% when the specificity ≤95%, and vice versa [26]. The prevalence of lung cancer in high-risk populations is 1 to 3%, while LDCT has about 90% sensitivity and only 61% specificity, which is prone to produce a high false-positive rate. The ideal prediction model should have > 95% specificity and appropriate sensitivity for identifying malignant PNs, thus could augment the performance of LDCT for lung cancer screening [27]. Our result appears promising; the developed prediction model achieved a sensitivity of 87.3% and a specificity of 95.7% with an AUC value of 0.951 in malignant PNs diagnosis, which suggested that the prediction model does possess the required diagnostic performance for routine clinical application.

However, our study also has some limitations. The sample size is small. The exact number of subjects in some histological subtype groups, such as small cell lung cancer, may be insufficient. A large sample size is needed in further studies to confirm the results. Furthermore, subjects in this study were recruited from hospital-based patients with PNs. The subjects might not be representative of a population-based LDCT screening setting for lung cancer. We will conduct a large trial of population-based LDCT screening to confirm the prediction model’s performance in identifying malignant PNs.

## Conclusions

In summary, we developed a simple prediction model based on DNA methylation biomarkers with radiological characteristics that could identify malignant from benign nodules detected by LDCT. Future use of the prediction model could reduce costs and avoid invasive diagnostic procedures for patients with benign PNs, at the same time, allowing immediate treatment for lung cancer patients. This prediction model could be used in combination with LDCT to improve the over-all diagnosis of lung cancer. Nevertheless, undertaking a prospective study of the prediction model for malignant PNs in an extensive population-based LDCT screening is required.

## Supplementary Information

**Additional file 1.**

## Data Availability

The datasets used and analyzed during the current study are available from the corresponding author on reasonable request. Subjects data are not publicly available for containing information that could compromise participants’ consent and confidentiality.

## Abbreviations

LDCT: Low-dose spiral computed tomography
LC: Lung cancer
PNs: Pulmonary nodules
PTGER4: Prostaglandin E receptor 4
RASSF1A: Ras association domain family 1A
SHOX2: Short stature homeobox gene two
ROC: Receiver operating characteristic
AUC: Area under a receiver operator characteristic curve
CI: Confidence interval
